# Association between antibiotics and gut microbiome dysbiosis in children: systematic review and meta-analysis

**DOI:** 10.1080/19490976.2020.1870402

**Published:** 2021-03-02

**Authors:** Lucy McDonnell, Alexander Gilkes, Mark Ashworth, Victoria Rowland, Timothy Hugh Harries, David Armstrong, Patrick White

**Affiliations:** School of Population Health and Environmental Sciences, King’s College London, London, UK

**Keywords:** Gut dysbiosis, Antibiotics, Children

## Abstract

Antibiotics in childhood have been linked with diseases including asthma, juvenile arthritis, type 1 diabetes, Crohn’s disease and mental illness. The underlying mechanisms are thought related to dysbiosis of the gut microbiome. We conducted a systematic review of the association between antibiotics and disruption of the pediatric gut microbiome. Searches used MEDLINE, EMBASE and Web of Science. Eligible studies: association between antibiotics and gut microbiome dysbiosis; children 0–18 years; molecular techniques of assessment; outcomes of microbiome richness, diversity or composition. Quality assessed by Newcastle–Ottawa Scale or Cochrane Risk of Bias Tool. Meta-analysis where possible. A total of 4,668 publications identified: 12 in final analysis (5 randomized controlled trials (RCTs), 5 cohort studies, 2 cross-sectional studies). Microbiome richness was measured in 3 studies, species diversity in 6, and species composition in 10. Quality of evidence was good or fair. 5 studies found a significant reduction in diversity and 3 a significant reduction in richness. Macrolide exposure was associated with reduced richness for twice as long as penicillin. Significant reductions were seen in *Bifidobacteria* (5 studies) and *Lactobacillus* (2 studies), and significant increases in Proteobacteria such as *E. coli* (4 studies). A meta-analysis of RCTs of the effect of macrolide (azithromycin) exposure on the gut microbiome found a significant reduction in alpha-diversity (Shannon index: mean difference −0.86 (95% CI −1.59, −0.13). Antibiotic exposure was associated with reduced microbiome diversity and richness, and with changes in bacterial abundance. The potential for dysbiosis in the microbiome should be taken into account when prescribing antibiotics for children.

Systematic review registration number: CRD42018094188

## Introduction

Research over recent years has emphasized the importance of the gut microbiome, and its association with health and the immune system. On the one hand, methods of enhancing the microbiome have proved effective. For example, probiotics have been used to reduce the incidence of severe necrotizing enterocolitis in preterm neonates as the gut microbiome is insufficiently developed to regulate the intestinal mucosa,^1^ and fecal microbial transplant (FMT) is being used successfully to treat patients with allergic colitis or *Clostridium difficile* infection.^2,3^ On the other hand, damage to the microbiome has been linked with conditions such as asthma,^4–7^ allergy,^8^ juvenile idiopathic arthritis,^9,10^ type 1 diabetes,^11^ obesity,^12–17^ celiac disease,^18^ mental illness,^19^ Crohn’s disease,^20^ and impaired neurocognitive outcomes.^21^

Although the mechanism of association for these diseases has not been fully explored, antibiotics, one of the most commonly prescribed drugs in children in western populations,^22^ appear to disrupt the normal maturation of the microbiome and destabilize it, altering basic physiological equilibria.^23,24^ Antibiotics also seem to affect gene expression, protein activity and overall metabolism of the gut microbiota which may directly influence major organ development and immune functioning.^25^ Antibiotic exposure has already been shown to alter the gut microbiome in adults and in neonates.^26,27^ This review sought to systematically examine the research into the association between antibiotic exposure and pediatric gut microbiome disruption.

## Results

### Study selection

**Figure 1.**
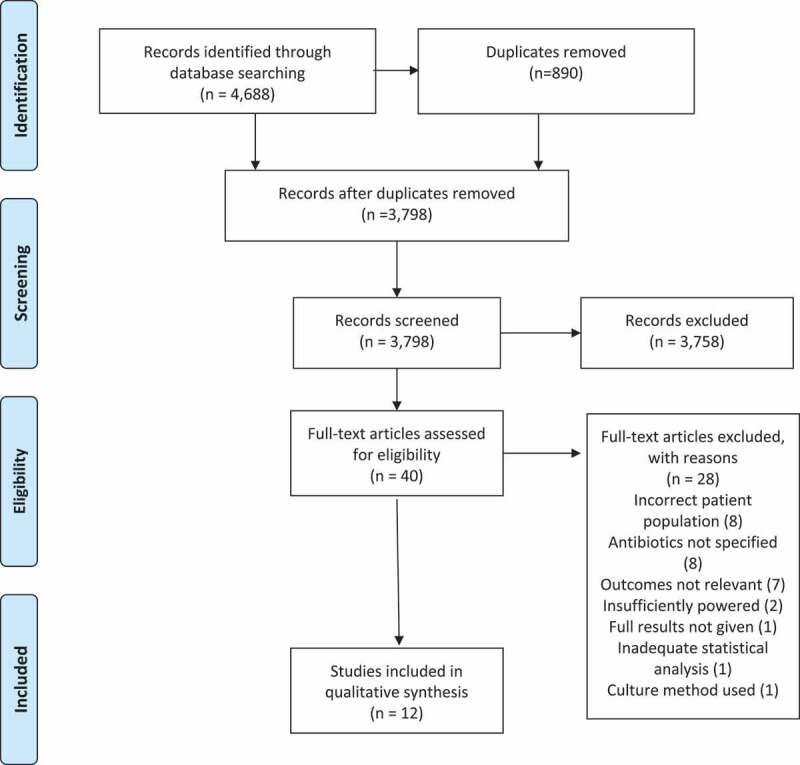
PRISMA flow chart. Preferred reporting items for systematic reviews and meta-analyses 2009

The literature search identified 4,688 publications. The process of publication selection is described in Figure 1. Twelve studies met the eligibility criteria, were deemed good (nine studies) or fair (3 studies) in quality and were included in the final analysis. Meta-analysis was carried out on four RCTs that shared the Shannon Index as their outcome measure of the impact of antibiotics up to 14 days after administration. Quality assessments of RCTs are presented in Supplementary Data Figure S1 (Cochrane Risk of Bias Tool).^28^A high risk of bias was found in Wei et al.’s trial with respect to blinding of the outcome in the analysis done at 4 years but there was no such risk with respect to the analysis done at 14 days.^29^Quality assessments (Newcastle–Ottawa Scale) of observational cohort studies are presented in Supplementary Data Table S1 and of cross-sectional studies in Supplementary Data Table S2.^30^,^31^

### Included studies’ design and participant characteristics

The main characteristics of the included studies are summarized in Table 1. There were five randomized controlled trials (RCTs), five cohort studies and two cross-sectional studies. All studies detected changes in composition of the microbiome following antibiotic exposure in 3 main outcomes: reduction in microbiome species richness; reduction in species diversity; and change in taxonomic composition (change in a specific phylum, genus or species). The primary aim varied between studies. The age of participants ranged from new-born to 12 years old. Nearly all studies reported the short-term associations between antibiotic exposure (up to 1 month) and microbiome composition; some also reported longer-term outcomes up to 2 years and 4 years.^33,37,29,42^

**Table 1.** Summary of included studies: author, year, location, design, aim, participants, antibiotic, duration, molecular techniques used, and outcomes

Author, year, location	Design and reference group	Aim	Study participants (n), age	Antibiotic exposure (route; duration)	Exposure to outcome interval (sampling frequency)	Molecular technique	Key findings: Richness	Key findings: Diversity	Key findings: Taxonomical change
1.Bai et al.^32^ China	Cross-sectional with (control group)	Added impact of antibiotics on microbiota changes in ALL	33 healthy children; 10 received antibiotics 1–12 years	Cephalosporin Penicillin (oral + IV: 10 days)	1–4 weeks (x1)	Next generation sequencing (NGS)	n/a	**Reduction in α-diversity** Shannon index, ~2.75 (antibiotics) vs ~3.25 (control), *p* < .05 ^#^ Simpson index ~0.15 (antibiotics) vs ~0.09 (control) *p* < .05 ^# a^	**Decreased** *Firmicutes/Bacteroidetes* ratio by approximately one third (*p* < .05) **No difference** in abundances of Actinobacteria and Proteobacteria
2. Bokulich et al,^33^ USA	Cohort (baseline assessment)	Microbiome development in first 2 years of life	43 infants, 25 received antibiotics 0–2 years	Cephalosporin Beta-lactams Macrolides Quinolones Nitrofurantoin (route/duration not specified)	3–139 days (x25)	PCR, 16s RNA gene amplification	n/a	**No change in α-divers**ity after antibiotic exposure for median 52 days (13–139) **Reduced β–diversity**: UniFrac distance, Permutational MANOVA, R^2^ < 0.01, *p* < .001	**No effect** on *Bifidobacterium* abundance
3.Brunser et al.^34^ Chile	RCT (baseline assessment)	Impact on microbiome of prebiotic supplement following antibiotic	130 infants, before and after antibiotics 1–2 years	Amoxicillin (oral; 7 days)	1–3 weeks (x3)	FISH* and flow cytometry	n/a	n/a	**Decrease** in amoxicillin-associated fecal bacteria by 30% (*p* < .001) **Increase** in amoxicillin associated *E. Coli* Log count 4.77 ± 0.96 (baseline) vs 5.10 ± 1.39 after treatment (*p* = .015) **No change** in total counts of *Bifidobacterium* and *Bacteroides*
4.Doan et al.^35^ Niger	RCT (control group)	Effects of azithromycin on gut microbiome diversity	80 children, 40 received antibiotics 1–5 years	Azithromycin (oral; single dose)	5 days (x1)	16S rRNA sequencing	n/a	**Reduction in α-diversity**: Inverse Simpson’s α-diversity decreased (5.03 95% CI 4.08–6.14) vs placebo (6.91; 5.82 − 8.21) *p* = .03 Shannon’s α-diversity decreased (10.60; 95% CI 8.82–12.36) vs placebo (15.42; 13.24–17.80) *p* = .004 **No change in β-diversity** **Decrease** in Simpson’s community level γ diversity with azithromycin (10.10 95% CI 7.80–11.40) vs placebo (17.72;13.80–20.21) (*p* < .001)	n/a
5.Fouhy et al.^36^ Ireland	Cohort study (control group)	Effect of antibiotics on gut microbiome	18 children, 9 received antibiotics Newborn	Ampicillin and gentamicin (IV:2–9 days)	4 and 8 weeks (x2)	High throughput sequencing of 16S rRNA	n/a	**No change in α-diversity** (Shannon Index) at 4 weeks (3.6) vs control (3.6) (*p* = .575)	**Decreased** *Bifidobacterium* (5% vs 25%; *p* = .013) and *Lactobacillus* (1% vs 4%; *p* < .009) in treated group at 4 weeks vs control; no difference at 8 weeks **Increased** *Proteobacteria* (44% vs 23%; *p* < .005) *and Enterobacteriaceae* (50% vs 32%; p = .006) at 8 weeks vs control. **Increased** Clostridium in treated infants than controls at week 8 (7% vs 2%, *p* < .035)
6.Korpela et al.^37^ Finland	Retro-spective controlled cohort study	Antibiotic induced changes in microbiota composition	142 children, 99 received antibiotics 2–7 years	Macrolides Penicillins (route/duration not specified)	Variable (<6 months to 2 years) (x1-2)	DNA extraction, 16s rRNA gene sequencing	Macrolides: **Reduced** richness up to 2 years (*p* < .05)^#^ Penicillins: **Reduced** richness over 6 months^#^ (*p* < .001)resolved by 12 months	n/a	Macrolides: Exposure over 6 months **Reduced** *Bifidobacterium* (0.23-fold change *p* < .004) and *Lactobacillus* (0.12-fold change *p* < .004) **Increased** *Bacteroides* (2.04-fold change *p* < .004) and Proteobacteria (1.96-fold change (*p* < .02). Penicillins: **Decreased** *Lactobacillus* (0.09 fold; *p* < .004) with exposure in previous 12 months
7.Mangin et al.^38^ Chile	Cohort (baseline assessment)	Impact of amoxicillin on fecal bifidobacteria	31 infants, all received antibiotics 12–24 months	Amoxicillin (oral: 7 days)	0 days (x2)	Total DNA extraction, PCR	n/a	n/a	**No change** in total *Bifidobacteria* **Disappearance** of *Bifidobacterium adolescentis* species (0% vs 36.4% (*p* < .001)
8. Oldenburg et al.^39^ Burkina Faso	RCT (baseline assessment)	Investigate effect of 3 antibiotics on microbial diversity	124 children, 93 received antibiotics 6–59 months	Amoxicillin Azithromycin Cotrimoxazole (oral: 5 days)	5 days (x2)	DNA extraction, deep gene sequencing	n/a	**Reduced α- diversity** with Azithromycin: Inverse Simpsons’ index decreased (6.6 95% CI 5.5–7.8) vs baseline (8.8 95% CI 7.5–10.1) (*p* < .001) Shannon index decreased (11.0 95% CI 9.3–12.7) vs baseline (14.6 95% CI 13.0–16.2) (*p* < .001) **No reduction** with Amoxicillin or Cotrimoxazole	n/a
9.Parker et al.^40^ India	RCT (baseline assessment)	Assess microbiota changes following azithromycin	114 infants, 56 received antibiotics 6–11 months	Azithromycin (oral: 3 days)	12 days (x2)	16s rRNA gene sequencing, DNA extraction PCR	**Lower OTU** with azithromycin: (68.1 ± 15.4) vs placebo (73.6 ± 13.7) (linear regression *p* = .027) c. 7% less	**No significant change in α-diversity** (Shannon index) azithromycin (2.6 95%CI 2.47–2.73) vs placebo (2.8 95%CI 2.8(2.67–2.93) (*p* = .087)	**Decreased** relative abundance of Proteobacteria (mainly *Escherichia)* mean % ± SD: 15.9 ± 13.2 vs 10.2 ± 15.4 FDR (*p* < .001) and Verrucomicrobia (genus *Akkermansia muciniphilia)* 0.5 ± 3.1 vs 0.0 ± 0.0 FDR (*p* < .012) **No change** in Actinobacteria, Bacteroides and Firmicutes, *Bifidobacterium*
10.Penders et al.^41^ The Netherlands	Cross Sectional (control group)	Examine contribution of external influences to gut microbiota composition	1032 infants, 28 received antibiotics 1 month	Mainly Amoxicillin (oral: duration not specified)	<1 month (x1)	DNA isolation, PCR	n/a	n/a	**Decrease** with antibiotics in *Bifidobacteria*. Antibiotics (10.29 CFU/g log10) vs *Control* (10.7 CFU/g log10 (*p* < .01) **Decrease** in *Bacteroides fragilis*. Antibiotics (6.39 CFU/g log10) vs Control (9.31 CFU/g log10) (*p* < .01) **No change** in *Lactobacilli, Escherichia coli, Clostridium difficile*
11. Wei et al.^29^ Denmark	RCT (control group)	Examine short- and long-term impacts of azithromycin treatment on gut microbiota in children	72 children, 33 episodes of asthma-like symptoms received antibiotics 12–36 months	Azithromycin (oral: 3 days)	14 days and up to 4 years (x3)	DNA extraction and sequencing	**Decrease** in richness at 14 days: Observed richness: 23% reduction (177.8 ± 56.0 vs. 230.6 ± 61.2, *p* < .001); no difference by mean 233 days	**Reduced α-diversity**: at 14 days: Shannon index: 13% reduction; 2.96 ± 0.80 (mean ± SD) vs control 3.41 ± 0.58, *p* = .009) **Reduced β–diversity**: UniFrac distance, treatment accounted for variance (R^2^ = 3.8%, *p* = .027 (weighted) and F2 = 4.2% *p* < .001 (un-weighted)	**Reduction**, 50-fold, in genus *Bifidobacterium* at 14 days (p adjusted <0.011 (FDR *p* < .05) Long term (13–39 months) no differences seen between azithromycin and placebo groups
12.Yassour et al.^42^ Finland	Cohort (control group)	Study development of infant gut microbiome and effect of antibiotics	39 children, 20 received antibiotics 2–36 months	Amoxicillin Cefalexin Clarithromycin Amoxicillin and clavulanic acid Trimethoprim and sulfadiazine Azithromycin Cefaclor Penicillin G Netilmicin (oral: duration not specified)	<1 month (x28)	16S rRNA gene and whole genome shotgun sequencing	n/a	**Reduced** microbiome strain (subspecies) diversity (diversity index 0.0003 vs 0.55 (control) (*p* < .001)	**Decreased** abundance of species from *clostridium* clusters IV and XIVa (T regulatory immune cells) at aged 3 (median abundance ~9% vs ~15% control)^b^ (*p* = .037) Less stable gut microbiome following antibiotic treatment (Jaccard Index *P* = <0.001)

**Key**: # – Approximate mean values taken from a Box and Whisker plot; ꝉ – statistical significance testing, confidence intervals, or standard deviations not given; a – higher the Simpson index, the lower the diversity; b -raw data not given, approximate values taken from graph

**Abbreviations**: n/a- data not available. ALL: acute lymphoblastic leukemia. CFU/g = Colony forming units per gram of sample. FISH: Fluorescent in-situ hybridization. FDR – False Discovery Rate correction. OTU count: operational taxonomical unit.

### Microbiome richness

Microbiome richness (Table 6) data were available for 3 studies and are shown in Table 2. Microbiota richness in children exposed to antibiotics was statistically significantly reduced compared to that of children not exposed to antibiotics in all three studies.^29,37,40^ Measures of richness included Operational Taxonomic Unit (OTU) count (see Table 2) and a generic measure of ‘observed richness’. The time between exposure and analysis was ≤ 14 days in 2 studies,^29,40^ and ≤ 6 months in one study.^37^ The reduction in richness reported by Wei et al. had resolved by the time of a second analysis (mean of 223 days following exposure).^29^ Korpela et al., found that microbiome richness was reduced for up to 1 year following penicillin exposure and for up to 2 years following macrolide exposure.^37^ Parker reported that the significant reduction in species richness was driven through depletion of Proteobacteria (mainly the species *Akkermansia mucinophilia)* which were particularly susceptible to azithromycin.^40^ Three other authors also commented on richness but did not report raw data and hence are not included in Table 2.^32,36,42^

**Table 2.** Associations between antibiotic use and changes in microbiome richness in children up to 7 years

Study	Type	Age group	Country	Antibiotic	Duration of treatment	Time from exposure to analysis	Index of richness used	Placebo or Control (mean ± SD)	Intervention (mean ± SD)	Percentage difference	Significance
Wei et al.^29^	RCT	1–3 years	Denmark	Azithromycin	3 days	14 days	Observed richness	230.6 ± 61.2	177.8 ± 56.0	−25.9%	*p* < .001
Parker et al.^40^	RCT	6–11 months	India	Azithromycin	3 days	12 days	OTU count	73.6 ± 13.6	68.1 ± 15.4	−7.5%	*p* = .027
Korpela et al.^37^	Retro- spective cohort	2–7 years	Finland	Macrolides (M) Penicillins (P)	n/a	<6 months	OTU count	230^#^	175^#^ (M) 180^#^ (P)	−23.91%(M) -21.74% (P)	*p* < .001 *p* < .001

# Approximate value taken from bar chart. Confidence intervals or standard deviations not available. n/a = not available.

SD = Standard Deviation.

### Microbiome diversity

**Figure 2.**
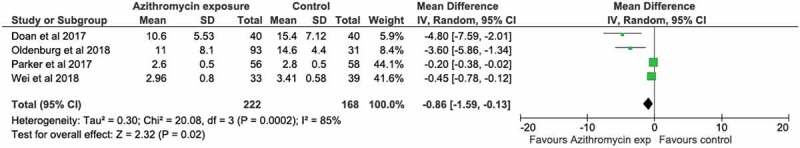
Meta-analysis of trials of azithromycin that used Shannon Index of microbiome alpha diversity as the outcome

Microbiome species diversity was reported by 8 studies.^32,33,35,36,39,40,29,42^ Data were available for 6.^29,32,35,36,39,39,40^ The main diversity outcome measure was alpha-diversity (Table 3). Antibiotic use was associated with a reduction in alpha-diversity (measured by Shannon or Simpson/Inverse Simpson indices) in 4 studies.^29,32,35,39^ Initial Shannon diversity indices varied substantially by geographical location (approximate index value of ‘3ʹ in studies in China, Denmark, India, and Ireland to approximate index value of ‘15ʹ in Burkina Faso and Niger). We carried out a meta-analysis of 4 RCTs examining the effect of azithromycin on the microbiome measured by the Shannon Index. We found a statistically significant overall reduction in alpha-diversity (mean difference −0.86 (−1.59 to −0.13, *p* < .001) (Figure 2).

**Table 3.** Association between antibiotic use and changes in microbiome alpha-diversity (species level) in children up to 12 years

Study authors, year, type and setting	Age group	Antibiotic	Days of therapy	Time between exposure and analysis	Indices of alpha-diversity used	Placebo/Control Group mean (95% CI)	Intervention group mean (95% CI or SD)	Percentage difference	Significance
Doan et al.^35^ RCT Niger	1–5 years	Azithromycin	Single dose	5 days	Shannon Inverse Simpson	15.42 (13.24–17.80) 6.91 (5.82–8.21)	10.60 (8.82–12.36) 5.03 (4.08–6.14)	−31.25% -27.21%	*p* = .004 *p* = .03
Oldenburg et al.^39^ RCT Burkino Faso	6–59 months	Azithromycin	5 days	5 days	Shannon Inverse Simpson	14.6 (13.0–16.2) 8.80 (7.5–10.1)	11.0 (9.3–12.7) 6.6 (5.5–7.8)	−24.65% -25.0%	*p* < .001 *p* < .001
Wei et al.^29^ RCT Denmark	1–3 years	Azithromycin	3 days	14 days	Shannon	3.41 (3.23–3.59)	2.96 (2.69–3.23)	−13.19%	*P* = .009
Parker et al.^40^ RCT India	6–11 months	Azithromycin	3 days	12 days	Shannon	2.8 (2.67–2.93)	2.6 (2.47–2.73)	−7.14%	*P* = .087
Bai et al.^32^ Cross-sectional China	1–12 years	Cephalosporin, Penicillin	10 days	1–4 weeks	Shannon Simpson	3.25*	2.75*	−15.38%	*P* < .05
Fouhy et al.^36^ Cohort Ireland	New-born	Ampicillin; gentamicin	2–9 days	4 weeks	Shannon	3.8^†^	3.6^†^	−5.26%	*P* = .575

† Confidence intervals or standard deviation not available*approximate values taken from box and whisker plot.

Beta-diversity was reported in 3 studies and significantly reduced in 2 of those.^29,33,35^ Bokulich examined the impact of exposure to several different classes of antibiotics: cephalosporins, beta-lactams, macrolides, quinolones, and nitrofurantoin.^33^ They found that although microbiome alpha-diversity was unchanged following antibiotic exposure, beta-diversity differed significantly between children exposed to antibiotics and those unexposed (UniFrac distance, permutational MANOVA, R^2^ < 0.01 *p* < .001).^33^ This means that although the individual diversity index did not change (i.e. wide species variety and abundance) there was a significant change in the types of species found. With regards to azithromycin exposure alone, Wei reported associations with reduced alpha and beta diversity.^29^ Doan however found that beta diversity was unaffected (i.e., similar types of species in the two groups) following azithromycin exposure. But there was a 43% decrease in Simpson’s community-level gamma diversity (*p* < .001) which reflected the overall reduction in bacterial diversity of the treatment group compared to the placebo group.^35^ Six of eight studies reporting on species diversity found a significant association between antibiotic use and a reduction in species diversity.

### Taxonomic composition

The major phyla reported in all studies were Actinobacteria, Bacteroidetes, Firmicutes, and Proteobacteria. One study reported the phylum Veruccomicrobia.^40^ A significant increase or decrease in the abundance of a particular phylum, genus or species was reported in 10 studies. These results are summarized in Table 4.

**Table 4.** Associations between antibiotic use and changes in taxonomic composition of the microbiome in children up to 12 years

Study Author, year, type of study, country, duration.	Antibiotic (days of treatment where given)	Time from exposure to analysis	Actinobacteria (includes genus Bifidobacteria)	Bacteroidetes	Firmicutes (gram positive)	Proteobacteria (gram negative)
Bai et al.^32^ Cross-Sectional China 1–12 years	Cephalosporin, Penicillin 10 days	1–4 weeks	No change in Actinobacteria	F/B ratio decreased by approx. 1/3(*p* < .05) * (increase in Bacteroidetes)	F/B ratio decreased by approx. 1/3 (*p* < .05) * (decrease in Firmicutes)	No change
Bokulich et al.^33^ Cohort USA 0–2 years	Cephalosporin Beta lactams Macrolides Quinolones Nitrofurantoin	3–139 days	No change in *Bifidobacteria*	Increased (*p* < .05) *	Clostridiales decreased from 3–9 months of age*	Increased (*p* < .05) *
Brunser et al.^34^ Cohort Chile 0–2 years	Amoxicillin 7 days	1–3 weeks	No change in *Bifidobacteria*	No change	n/a	*E. coli* increased vs baseline (Log 4.77 ± 0.96 vs Log 5.10 ± 1.39 *p* = .015)
Fouhy et al.^36^ Cohort Ireland Neonates	Ampicillin; gentamicin 2–9 days	4 weeks	Lower levels of *Bifidobacteria* at 4 weeks vs control (5% vs 25%; *p* = .013); no difference at 8 weeks	No change	Lower levels of *Lactobacillus* at 4 weeks vs control (1% vs 4%; *p* < .009); no difference at 8 weeks	Higher proportions of Proteobacteria (44% vs 23%; *p* < .005) and *Enterobacteriaceae* (50% vs 32%; *p* = .006) at 8 weeks vs control
Korpela et al.^37^ Retrospective cohort Finland 2–7 years	Macrolides (M) Penicillins (P)	<6 months	Exposure in previous 6 months M: *Bifidobacterium* (0.23-fold decrease *p* < .004) P: *Bifidobacterium*: no change	Exposure in previous 6 months M: *Bacteroides* increased (2.04-fold change *p* < .004) P: Bacteroides: no change	Exposure in previous 6 months M*: Lactobacillus* decreased (0.12-fold change *p* < .004), Clostridium increased (2.68-fold change *p* < .004) P: *Lactobacillus* decreased (0.11-fold change *p* < .004), Clostridium: no change	M: Proteobacteria increased (1.96-fold change *p* < .02) with exposure in previous 6 months P: no change
Mangin et al.^38^ Cohort Chile 6–59 months	Amoxicillin 7 days	0 days	Total *Bifidobacterium* concentrations not significantly altered but complete disappearance of *Bifidobacterium adolescentis* species (0% vs 36.4% (*p* < .001)	n/a	n/a	n/a
Parker et al.^40^ RCT India 6–11 months	Azithromycin 3 days	12 days	No change in Actinobacteria or *Bifidobacterium*	No change	No change	Proteobacteria reduced (relative abundance mean % ± SD: 15.889 ±13.207 (placebo) vs 10.200± 15.401 (azithromycin), FDR *p* < .001) *E.coli* reduced (relative abundance mean % ± SD:12.087± 12.457 (placebo) vs 7.309 ±13.258 (azithromycin), FDR *p* < .013)
Penders et al.^41^ Cross sectional Netherlands 1 month	Amoxicillin**	< 1 month	*Bifidobacteria* median count placebo log10 CFU/g feces 10.70 vs amoxicillin log CFU/g feces 10.29 (*p* < .01)	*Bacteroides fragilis* median count placebo log10 CFU/g feces 9.31 vs amoxicillin log10 CFU/g 6.39 (*P* < .01)	No change (lactobacilli)	No change in *Clostridium difficile* and *E.Coli*
Wei et al.^29^ RCT Denmark 1–3 years	Azithromycin 3 days	14 days	50 x reduction (fold change) in genus *Bifidobacterium* at 14 days (*p* adjusted < 0.011 (FDR *p* < .05)	n/a	n/a	n/a
Yassour et al.^42^ Cohort Finland 2–36 months	Amoxicillin Cefalexin Clarithromycin Amoxicillin and clavulanic acid Trimethoprim and sulfadiazine Azithromycin Cefaclor Penicillin G Netilmicin	< 1 month	n/a	n/a	Decrease in abundance of species from clostridium clusters and 14XIVa IV (T regulatory immune cells) at aged 3 (median abundance ~9% vs ~15% control) ^b^ (*p* = .037)	n/a

**Key**: †Confidence intervals or Standard Deviation not available. # Approximate value taken from bar chart. No confidence intervals or SD available. *no raw data. ** authors state that the antibiotics taken were ‘mainly amoxicillin’. ^b^ raw data not given; approximate values taken from graph.

**Abbreviations**: CFU/g = Colony forming unit/gram; F/B ratio = Firmicutes/Bacteroidetes ratio. FDR – false discovery rate correction. M = macrolides . *P* = penicillins. n/a = Data not available.

#### Actinobacteria

The association between antibiotics and the abundance of genus *Bifidobacterium* (phylum Actinobacteria) was examined in 9 studies (Table 4). In five studies, antibiotics were significantly associated with reduced abundance of *Bifidobacteria*.^29,36–38,41^ Both penicillins and macrolides were associated with a decrease in *Bifidobacteria* although in some studies there was no change. Comparing macrolides with penicillins, Korpela et al. found that exposure to macrolides was associated with a fourfold decrease in *Bifidobacteria* but that exposure to penicillins was not associated with *Bifidobacteria* levels.^37^ Fouhy et al. found that a combination of ampicillin and gentamicin was associated with reduced *Bifidobacteria* initially, but that by 8 weeks levels had returned to that of the control group.^36^ At species level, Mangin et al. found amoxicillin exposure was associated with complete disappearance of *Bifidobacterium adolescentis* but that overall concentrations of *Bifidobacteria* were not altered.^38^

#### Bacteroidetes

The association between antibiotics and the abundance of Bacteroidetes phylum (which includes the genus *Bacteroides*) was examined in seven studies (Table 4). There was a statistically significant change in 4 studies. The 3 studies that reported an increase in Bacteroidetes examined exposure to a combination of antibiotics including cephalosporins and macrolides.^32,33,37^ One study examining only amoxicillin exposure reported a decrease of the species *Bacteroides fragilis*.^41^ In 3 studies there was no change (studies examining amoxicillin, ampicillin/gentamicin and azithromycin).^34,36,40^

#### Firmicutes

The association between antibiotics and the abundance of Firmicutes phylum (which includes the genera *Lactobacillus* and *Clostridium*) was examined in seven studies (Table 4).^32,33,36,40,41,42^ A statistically significant decrease was seen in 4 studies following antibiotic exposure.^32,36,37,42^ Korpela et al. reported that *Lactobacillus* levels were reduced for up to 12 months following penicillin use and for up to 24 months following macrolide use.^37^ The same study found a nearly 3-fold increase in *Clostridium* within 6 months of exposure to macrolides only (details of specific species not given).^37^ Yassour et al reported a 40% decrease in *Clostridium spp*. belonging to clusters IV and XIVa (inducers of T regulatory immune cells) in children aged 3 who had had antibiotics.^42^

#### Proteobacteria

The abundance of Proteobacteria following antibiotic exposure was examined in six studies (Table 4). In 5 studies there was a statistically significant change in Proteobacteria following exposure to a variety of antibiotics, however the direction of association was not consistent. At phylum level, 4 studies reported an increase in Proteobacteria following exposure to different antibiotics including penicillins, cephalosporins and macrolides.^33,34,36,37^ One study reported a decrease in Proteobacteria following azithromycin exposure only.^40^ At species level, a statistically significant increase in *E.coli* was reported following amoxicillin exposure in children aged 1–2 years,^34^ but a statistically significant decrease in *E.coli* was reported following azithromycin exposure in children aged 6–11 months.^40^

#### Verrucomicrobia

The association between azithromycin and a reduction in the abundance of phylum Verrucomicrobia was examined in one study (Table 4). This phylum has relatively few species described. Parker et al. examined the association between azithromycin and the species *Akkermansia mucinophila* which completely disappeared with azithromycin use (*p* < .003).^40^

## Discussion

### Key findings

As far as we are aware this is the first systematic review to synthesize the evidence of the association between antibiotic exposure and changes in the microbiome specifically in children. We found evidence of microbiome disruption characterized by changes in richness, diversity, and taxonomic composition. We cannot be sure of the duration of these changes from the data presented as most studies only presented short-term data. The studies were heterogeneous, with variation between studies in participant age, setting, duration of antibiotic exposure, type of antibiotic given, mode of delivery, outcome measures and time between exposure and analysis. These factors may influence the association between antibiotic use and microbiome composition. Evidence of change in a wide range of microbiome characteristics associated with antibiotic exposure requires further investigation and explanation.

We found evidence that antibiotic exposure was associated with a reduction in both richness and diversity. In particular azithromycin exposure reduced microbiome alpha-diversity by a mean reduction in Shannon index of 0.86. The studies looked at a variety of antibiotics covering narrow to broad-spectrum antibiotics, with macrolides and penicillins representing the antibiotics most commonly studied. Although no specific change in richness or diversity emerged according to antibiotic class, we found evidence that macrolides were associated with more changes in the microbiome than penicillins and with effects that persisted for longer.^37,39^

We also found evidence that antibiotic use was associated with a reduced number of gut bacteria thought to be beneficial. *Bifidobacteria* (phylum Actinobacteria) and *Lactobacilli* (phylum Firmicutes) are producers of short-chain fatty acids which have positive effects on mammalian energy metabolism and form the basis of probiotic supplements.^45^ The majority of studies, however, did not report changes in these genera at species level which limits our appreciation of the changes in specific species associated with antibiotics. We also found evidence that changes in other beneficial bacteria were associated with antibiotic use. One study reported a decrease in Clostridium clusters IV and XIVa which are inducers of T regulatory immune cells which have a role in regulating or suppressing other cells in the immune system.^42^ A second found that Azithromycin was statistically significantly associated with reduced numbers of *Akkermansia Mucinophilia*.^40^ This species has previously been recognized as having anti-inflammatory and immunostimulant properties, and improving intestinal barrier function, endotoxinaemia and insulin sensitivity.^46^

We found evidence that antibiotics were associated with a rise in Bacteroidetes and Proteobacteria following antibiotic exposure. These phyla include species which have been implicated in serious infection. Although *Bacteroides spp*. may provide some level of protection from invasive pathogens as a gut commensal, *Bacteroides* have also been associated with bloodstream infections and abscess formation.^47^ However, it cannot be assumed that higher levels of Bacteroides in the gut are the source of these infections. *E.coli* (Proteobacteria) is a common cause of urinary tract infections and sepsis and a major source of antimicrobial resistance.^48^

### Study strengths and limitations

Our review highlights important findings regarding the relationship between antibiotic exposure and microbiome disruption in children. A strength of our study is that we only included studies with named antibiotics which included specific details of antibiotic administration, rather than exposure to ‘antibiotics’ in general. However, several studies included more than one named antibiotic, so in these cases it was not possible to associate a particular change with a specific antibiotic or class. In the majority of studies, the indication for antibiotic use was infection. In one study there was no clinical indication for antibiotic use but associations with changes in the microbiome were still present. This supports the independent association between antibiotic exposure and microbiome disruption, although further studies of this relationship are required.^40^0

The use of different outcome measures limited our ability to make comparisons between studies. Although the primary outcomes reported in the RCTs were similar, the applicability of the meta-analysis result may be limited by variation in initial Shannon index scores which in turn might reflect microbiome diversity by geographical location. We could find no evidence of agreement in the literature on the definition of a normal Shannon Index. This substantial difference in variation by geographical location does not seem to have been highlighted in the literature previously and may be worth further investigation. Outcomes in observational studies covered a number of indices of richness, diversity, and taxonomical changes which precluded meta-analysis of all studies. This variation is likely to reflect a lack of consensus among researchers about the most suitable outcome measures in addition to the complexity of the microbiome itself.

The majority of the studies included in the review focussed on microbiome changes over a short time following antibiotic prescription, i.e. less than 1 month. There was limited evidence therefore of the duration of the changes following exposure. Studies that examined effects over time, found that microbiome disruption lasted between 1 and 2 years,^29,37^ depending on the antibiotic studied. In this interval some children will receive a further course of antibiotics potentially disrupting microbiome recovery.^37,49^ Further studies are necessary to determine the duration of microbiome disruption.

### Comparison/relation to existing literature

A systematic review of antibiotic prescribing in neonates (up to 44 weeks gestational age) looked at the effects of antibiotics on the neonatal microbiome and similarly found that antibiotic exposure was associated with reduced gut microbial diversity and reduced colonization rates of protective commensal bacteria, although the quality of evidence was low.^27^ A study looking at the gut microbiota of adults also found that antibiotic exposure was associated with a decrease in beneficial bacteria such as *Bifidobacterium* and butyrate producers and an increase in Enterobactericae (phylum Proteobacteria). The majority of the changes lasted for approximately 45 days, but the microbiome had not fully recovered by 180 days.^26^ Studies in mice support the findings of more reduced diversity following macrolide exposure compared to amoxicillin exposure. Cumulative effects on the microbiome of multiple antibiotic courses, delayed microbiome maturation following antibiotics and fewer changes associated with narrow-spectrum antibiotics have all been observed.^50,51^

### Conclusion

In conclusion this review has gathered compelling evidence that antibiotic exposure in children is associated with a reduction in richness and/or diversity, and a change in the balance of species in the microbiome with reductions in the numbers of commensal bacteria thought to be beneficial. Studies that looked at the impact on the microbiome for more than 1 month were limited but there is evidence that antibiotics are associated with disruption to the microbiome for up to 2 years. Macrolide antibiotics cause immediate and longer term damage. More detailed understanding of the strength and duration of antibiotic-specific associations with microbiome dysbiosis in children is needed. Evidence should be sought of a causal relationship between antibiotic use in children, gut dysbiosis and subsequent risk of local or systemic pathological changes with repeated courses of antibiotics. In the meantime, healthcare practitioners should consider the potential for damage to the gut microbiome when prescribing antibiotics for children.

## Methods

Procedures used in this review were consistent with Preferred Reporting Items for Systematic Reviews and Meta-Analyses (PRISMA) guidelines.

### Protocol and registration

A review protocol was submitted in advance to PROSPERO, a database of systematic review protocols (registration ID: CRD42018094188).

### Eligibility criteria

Our inclusion criteria were: studies of any design-assessing change in the microbiome associated with named antibiotic exposure; participants aged from 0 to <18 years (excluding pre-term babies); assessment of composition and diversity of the microbiome using a genetic analysis technique; comparable reference group or baseline assessment and adequate statistical analysis. Our exclusion criteria were non-original research; studies investigating the impact of antibiotics in labor on neonates; studies investigating exposure to any intervention which was not a named antibiotic; studies assessing the impact of antibiotics on other systemic microflora only, e.g. skin, nasal; conference abstracts where insufficient data were given and where the study authors did not reply to further enquiries; and non-English language articles.

### Information sources and search strategy

The literature search was performed in February 2019. The databases searched were MEDLINE, EMBASE and Web of Science. No restrictions were placed on the publication period. Search terms included both text words and MESH terms. The full search strategy can be viewed in Supplementary data Table S3.

### Study Selection and data collection process

Papers were screened using Covidence software (Melbourne, Australia) to efficiently identify the most relevant and appropriate papers. The first reviewer (LM) conducted the literature search and imported the references. Duplicate articles were removed. Two reviewers (LM and AG) screened titles and abstracts with respect to eligibility criteria. Full-text articles of potentially relevant studies were independently assessed for eligibility by two reviewers (LM and VR). Any disagreements were reviewed by another reviewer (PW) and resolved through discussion.

### Data extraction

Information was extracted from included studies on the study type, purpose, characteristics of study participants (age, co-morbidities), details of the antibiotic exposure (name, route of administration), time between exposure and microbiome analysis, molecular technique used and study outcomes. Molecular techniques used included Fluorescent in-situ hybridization (FISH) and flow cytometry, 16s RNA sequencing, DNA extraction, Next Generation Sequencing (NGS) and whole-genome shotgun sequencing (see Table 5). We excluded papers that did not name the antibiotic as we could not guarantee that the participants had been exposed to antibiotics.

**Table 5.** Definition of molecular techniques used by studies in the review

Technique name	Definition
Fluorescent in-situ hybridization (FISH)	Molecular cytogenic analysis using fluorescent probes to detect, quantify and map genetic material.
Flow cytometry	Analysis of the frequency and other properties of cells stained with specific fluorochrome conjugated antibodies to identify bacteria, their viability, and their DNA content.
16s rRNA sequencing	Amplification of a piece of RNA (amplicon) and sequencing to identify and compare bacteria within a sample.
DNA extraction	Purification of DNA using physical and chemical methods
Next Generation Sequencing (NGS)	Sequencing of DNA and RNA with different technologies
Whole genome shotgun sequencing	Comprehensive sampling of all genes in all organisms present to evaluate diversity and study ‘difficult to culture’ microorganisms.

### Meta-analysis

eta-analysis was performed where studies shared the same outcome and where output data were available to include in the analysis. We performed a meta-analysis of four RCTs including 390 patients looking at the mean difference in Shannon Index before and after antibiotic exposure. Continuous outcomes were analyzed using an inverse variance model with a 95% CI. Values were reported as mean differences. *P*-values were two-tailed and statistically significant if *p* < .05. Statistical heterogeneity quantification was performed using the I^2^ statistic. Degrees of heterogeneity were defined as none (I^2^ 0–20%), low (I^2^ 25–49%), moderate (I^2^ 50–74.9%) and high (I^2^ > 75%). When heterogeneity was quantified as low or above, a random-effects model was used. The meta-analysis was performed using review manager (Revman) for MAC (Version 5.3. Copenhagen: The Nordic Cochrane Center. The Cochrane Collaboration, 2014).

### Quality assessment and risk of bias

Observational study quality (cohort and cross-sectional studies) was assessed using a modified version of the Newcastle–Ottawa scale.^30,31^ The Newcastle–Ottawa scale is used to assess quality and biases. Points are assigned on a nine-point scale. LM and PW independently assessed quality factors including: i) comparability of exposed and non-exposed groups; ii) evidence of microbiome assessment prior to exposure; iii) record of antibiotic exposure; iv) confounding factors; and v) statistical analysis. RCT quality was assessed using the Cochrane Risk of Bias Tool.^28^ LM and PW independently applied the risk of bias assessments to each RCT. Disagreement was resolved through discussion.

Additional quality features for RCTs included clear description of inclusion/exclusion criteria and of withdrawals/dropouts.

### Summary measures

The primary outcome measure was the change in bacterial composition of the microbiome. This was measured as the changes in microbiome richness, alpha-diversity or taxonomic composition.^29,33,35^ Secondary outcome measures were beta- and gamma-diversity.^29,33,35^ Microbiome richness score measures the total number of species found in a single sample. Microbiome alpha-diversity score measures the number of individual bacteria from each of the bacterial species isolated from a single sample. Beta-diversity examines the differences in species composition between 2 samples.^29,33,35^ Gamma-diversity measures diversity across many samples taking into account the different species found in each sample.^35^ With regards to change in taxonomic composition, the four main phyla reported were Actinobacteria, Bacteroidetes, Firmicutes and Proteobacteria. The various different indices used by authors to quantify these measures are summarized in Table 6.

**Table 6.** Definitions and examples of indices measuring microbiome richness and diversity

Measures	Definition and example indices
Species Richness*^43^*	Total number of bacterial species in sample Example indices: *Operational Taxonomic Unit (OTU) count* OTUs are organisms defined by similarity in DNA sequences, usually 97% *Observed Richness/Richness score* *Chao 1 score*
Alpha Diversity	The number of individual bacteria from each bacterial species present in sample Example indices: *Shannon Index* Simpson Index* Inverse Simpson index*
Beta diversity	Difference in microbial composition between two samples Example index: Weighted and unweighted *UniFrac distances* (a distance metric used for comparing microbial communities)^44^
Gamma diversity	The overall total species diversity of a range of samples (incorporating the range of different species found in each sample) Example index *Simpson’s community-level gamma diversity*

*Simpson’s Index is an inverse scale i.e. the higher the score the lower the diversity. It is therefore often reported as the Inverse Simpson Index so that higher scores indicate higher diversity.

## Supplementary Material

Supplemental Material
