# Supplementary material for: Association between antibiotics and gut microbiome dysbiosis in children: systematic review and meta-analysis
Source: Gut Microbes. 2021 Mar 2;13(1):1870402. doi: 10.1080/19490976.2020.1870402 (PMC7928022; doi:10.1080/19490976.2020.1870402)
Supplement: Supplemental Material [file KGMI_A_1870402_SM6604.zip › SUPPLEMENTARY/Supplementary Fig S1 Cochrane Risk of Bias Tool.pdf]

Figure S1. Risk of bias in RCTs of the effect of antibiotics on disruption of the microbiome in children. Cochrane Risk of Bias Tool

Low risk of bias: + Uncertain risk of bias: ? High risk of bias: –

	Random sequence generation (selection bias)	Allocation concealment (selection bias)	Blinding of participants and personnel (performance bias)	Blinding of outcome assessment (detection bias)	Incomplete outcome data (attrition bias)	Selective reporting (reporting bias)	Other bias
Brunser et al 2005	+	+	+	+	+	?	+
Parker et al 2017	+	+	+	+	+	+	+
Doan et al 2017	+	+	+	+	+	?	?
Wei et al 2018	+	+	+	–	+	+	?
Oldenbur et al 2018	+	+	+	+	+	?	+
