# Supplementary material for: Association between antibiotics and gut microbiome dysbiosis in children: systematic review and meta-analysis
Source: Gut Microbes. 2021 Mar 2;13(1):1870402. doi: 10.1080/19490976.2020.1870402 (PMC7928022; doi:10.1080/19490976.2020.1870402)
Supplement: Supplemental Material [file KGMI_A_1870402_SM6604.zip › SUPPLEMENTARY/Supplementary Table S3 Database search strategies.docx]

**Supplementary Table S3. Database search strategies for Medline, Embase and Web of Science.**

| **Database** | **Medline and Embase** |
| --- | --- |
| Publication period | No restrictions |
| Search strategy | 1. Exp. Child/preschool/or exp infant or exp Child/or exp Paediatrics or exp Adolescent 2. Paed* mp 3. Child*mp 4. Infant*mp 5. 1 or 2 or 3 or 4 6. Exp. Anti-bacterial Agents/ 7. Antibacterial.mp 8. Antibiotic*mp 9. Antimicrobial*mp 10. 6 or 7 or 8 or 9 11. Exp Microbiota/ 12. Microbiome.mp 13. Microbiota.mp 14. 11 or 12 or 13 15. 5 and 10 and 14 16. Exp Intestines/ or exp Bacteria/or exp Gastrointestinal Tract/or exp Gastrointestinal microbiome/ or exp Intestinal Mucosa/ 17. Gut.mp 18. 16 or 17 19. 15 and 18 |
| Articles retrieved- Medline | 640 |
| Articles retrieved– Embase | 3280 |

| **Database** | **Web of Science** |
| --- | --- |
| Publication period | All years, no limits |
| Search strategy | 1. Paed* OR Child* or Infant AND 2. Antibacterial OR antibiotic OR antimicrobial AND 3. Microbiota OR microbiome AND 4. Gut OR intestine OR gastro* |
| Articles retrieved | 768 |
