# Supplementary material for: Association between antibiotics and gut microbiome dysbiosis in children: systematic review and meta-analysis
Source: Gut Microbes. 2021 Mar 2;13(1):1870402. doi: 10.1080/19490976.2020.1870402 (PMC7928022; doi:10.1080/19490976.2020.1870402)
Supplement: Supplemental Material [file KGMI_A_1870402_SM6604.zip › SUPPLEMENTARY/Supplementary tables S1 and S2.docx]

**Table S1** **Newcastle-Ottawa scale: Quality assessment of cohort studies**

| **Quality assessment scale** | **Accepted criteria** | **Bokulich et al** | **Fouhy et al** | **Korpela et al** | **Mangin et al** | **Yassour et al** |
| --- | --- | --- | --- | --- | --- | --- |
| **Selection**   1. Representative- ness of the exposed cohort | Representative of child from general population | * | * | * | - | - |
| 1. Selection of non-exposed cohort | Selected from same population as exposed cohort | * | * | * | - | * |
| 1. Ascertainment of antibiotic exposure | Written records including name of antibiotic | * | * | * | * | * |
| 1. Demonstration that outcome of interest was not present at start of study | Evidence of microbiome assessment prior to exposure | * | - | - | * | * |
| **Comparability** of cohorts on basis of design, or analysis controlled for confounders (max 2 stars) | Study controls for age, mode of delivery, method of feeding or BMI | * | ** | * | * | * |
| **Outcome**   1. Assessment of outcome | Detailed microbiome assessment using molecular techniques | * | * | * | * | * |
| 1. Was follow up long enough for outcomes to occur | Follow up any time post exposure | * | * | * | * | * |
| 1. Adequacy of follow up of cohorts | Complete follow up for all subjects | * | * | * | * | * |
| Total (max 9) | | 8 | 8 | 7 | 6 | 7 |
| **Quality rating** | | **Good** | **Good** | **Good** | **Fair** | **Good** |

**Table S2 Adapted Newcastle-Ottawa scale: Quality assessment of cross-sectional studies**

| **Quality assessment scale** | **Accepted criteria** | **Bai et al** | **Penders et al** |
| --- | --- | --- | --- |
| **Selection**   1. Representativeness of the sample | Representative of children from general population | * | * |
| 1. Sample size | Greater than 15 participants | * | * |
| 1. Non-respondents | Description of non-respondents and characteristics established | - | - |
| 1. Ascertainment of exposure (max 2 stars) | Written records including name of the antibiotic and route of administration | ** | ** |
| **Comparability** of study group and control or placebo group.  (max 2 stars) | Study controls for age, mode of delivery, method of feeding or BMI | ** | * |
| **Outcome**   1. Assessment of outcome | Detailed microbiome assessment using molecular techniques | * | * |
| 1. Statistical test | Statistical test used to analyse the data is clearly described and appropriate | * | * |
| Total (max 9) | | 8 | 7 |
| **Quality rating** | | **Good** | **Good** |
